# Modification of phytosterol composition influences cotton fiber cell elongation and secondary cell wall deposition

**DOI:** 10.1186/s12870-019-1830-y

**Published:** 2019-05-20

**Authors:** Qi Niu, Kunling Tan, Zhenle Zang, Zhongyi Xiao, Kuijun Chen, Mingyu Hu, Ming Luo

**Affiliations:** Key Laboratory of Biotechnology and Crop Quality Improvement, Ministry of Agriculture/Biotechnology Research Center, Southwest University, Tiansheng Road 2, Beibei, Chongqing, 400716 People’s Republic of China

**Keywords:** Cotton fiber, Fiber elongation, GhSMT2–1, Phytosterol, Secondary cell wall

## Abstract

**Background:**

Cotton fiber is a single cell that arises from the epidermis of ovule. It is not only a main economic product of cotton, but an ideal material for studying on the growth and development of plant cell. Our previous study indicated that phytosterol content and the ratio of campesterol to sitosterol fluctuated regularly in cotton fiber development. However, what effects of modified phytosterol content and composition on the growth and development of cotton fiber cell is unknown. In this study, we overexpressed the *GhSMT2–1*, a cotton homologue of sterol C-24 methyltransferase 2 gene in transgenic upland cotton plants to modify phytosterol content and composition in fiber cells and investigated the changes on fiber elongation and secondary cell wall deposition.

**Results:**

*GhSMT2–1* overexpression led to changes of phytosterol content and the ratio of campesterol to sitosterol in fiber cell. At the rapid elongation stage of fiber cell, total phytosterol and sitosterol contents were increased while campesterol content was decreased in transgenic fibers when compared to control fibers. Accordingly, the ratio of campesterol to sitosterol declined strikingly. Simultaneously, the transgenic fibers were shorter and thicker than control fibers. Exogenous application of sitosterol or campesterol separately inhibited control fiber cell elongation in cotton ovule culture system in vitro. In addition, campesterol treatment partially rescued transgenic fiber elongation.

**Conclusion:**

These results elucidated that modification of phytosterol content and composition influenced fiber cell elongation and secondary cell wall formation. High sitosterol or low ratio of campesterol to sitosterol suppresses fiber elongation and/or promote secondary cell wall deposition. The roles of sitosterol and campesterol were discussed in fiber cell development. There might be a specific ratio of campesterol to sitosterol in different developmental stage of cotton fibers, in which GhSMT2–1 play an important role. Our study, at a certain degree, provides novel insights into the regulatory mechanisms of fiber cell development.

**Electronic supplementary material:**

The online version of this article (10.1186/s12870-019-1830-y) contains supplementary material, which is available to authorized users.

## Background

Cotton fibers are unicellular, linear structures that arise from the epidermis of ovule. Fiber development can be divided into four distinctive but overlapping growth stages: fiber initiation, cell elongation, secondary cell wall deposition, and fiber maturation. Fiber initiation is visible on the epidermal surface of ovules on the day of anthesis, and it is followed by cell elongation. Fibers elongation appears in the period of 3–20 days post anthesis (DPA), peaking at around 10 DPA. Secondary cell wall deposition occurs from 15 to 40 DPA, followed by fiber maturation from 40 to 50 DPA. Eventually, fiber cells reach 30–40 mm in length, with cellulose accounting for over 90% of their dry weight [1, 2]. Owing to their highly elongated structure and rich cellulose content, cotton fibers serve as an excellent system for studying cellulose biosynthesis and cell wall formation, as well as other fundamental aspects of plant cell elongation [2, 3]. Furthermore, fiber elongation and cell wall deposition greatly impact the length, strength, and fineness of fibers, which are the major factors determining lint yield and quality. Therefore, considerable studies in the past two decades have focused on understanding the regulatory mechanisms underlying fiber development. Among the progress that has been made, many reports have revealed that phytohormones such as brassinosteroids (BRs), ethylene, and auxin and their interactions play important roles in fiber development [3–12]. BRs are produced through complex biosynthetic pathways, and the functions of the intermediates (phytosterol) involved in their upstream pathway (sterol-specific pathway) remain unclear.

Sterols are isoprenoid-derived molecules. Higher plants usually synthesize thousands of isoprenoid-derived compounds, commonly referred to as phytosterols, of which sitosterol, campesterol, and stigmasterol are the predominant forms [13]. Campesterol is a precursor of the class of plant steroidal hormones called BRs. Sitosterol is an important component of the membrane lipid bilayer (especially in lipid rafts), which regulates membrane permeability and fluidity and the activity of membrane-bound proteins [14–19]. Therefore, phytosterols are involved in numerous processes of plant development, such as cell division, elongation, and cellulose synthesis [20–26]. During cotton fiber growth, especially in the process of rapid elongation, a substantial amount of sitosterol is needed to support the vigorous addition of plasma membrane. Meanwhile, the signal for elongation, such as BR signaling, must be strengthened to regulate fiber elongation. In fact, genes involved in phytosterol biosynthesis, such as *GhSMT2–1*, *GhSMT1*, *GhCYP51G1*, and *GhHYDRA1*, and those involved in BR synthesis or signaling are preferentially expressed in fiber cells, and their expression peaks at the stage of rapid fiber elongation [7, 8, 27–34]. Furthermore, phytosterol levels are at their highest, and the gene encoding the sterol carrier protein (*GhSCP2D*) highly expressed at the fiber elongation stage [4, 12]. Although on gene expression and biochemical aspects, phytosterols are recognized as important factors for the growth and development of cotton fibers, it is unclear what effect of phytosterol content and composition changes on fiber growth.

In phytosterol biosynthesis pathway, sterol C-24 methyltransferase 2 (SMT2) is a key enzyme controlling a branching point that defines two biosynthetic routes: one producing 24-methyl sterols such as campesterol, and the other producing 24-ethyl sterols like sitosterol and stigmasterol [13, 35]. Manipulations of SMT2 activity have been reported to result in altered amounts of sitosterol and campesterol [35–38]. In this study, we investigated the effects of phytosterol content and composition changes on fiber cell elongation and cell wall formation by using transgenic cotton plants in which the *GhSMT2–1* gene was overexpressed.

## Results

### Generation of transgenic cotton plants overexpressing *GhSMT2–1*

**Fig. 1:**
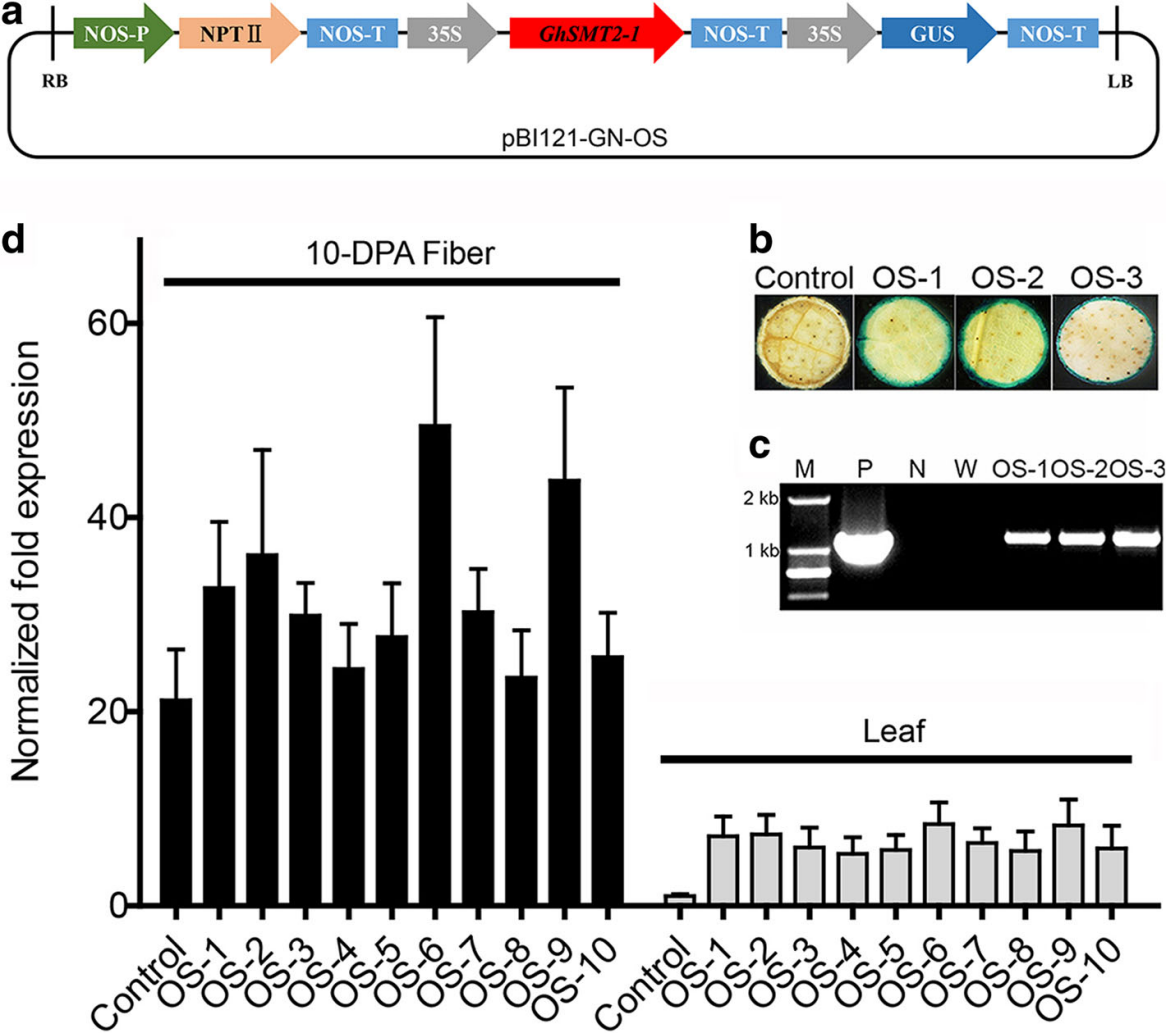
Sketch of expression vector and characterization of transgenic cotton plants overexpressing *GhSMT2–1*. **a** Sketch of pBI121-GN-OS. RB and LB is right border and left border of T-DNA region, respectively. NOS-P: NOS promoter; NOS-T: NOS terminator; 35S: CaMV 35S promoter. **b** GUS staining in cotton leaf discs. **c** Genotyping of transgenic cotton plants by PCR. A fragment of *GhSMT2–1* cDNA was amplified from genomic DNA. M, DNA marker; P, positive control, amplified from a plasmid containing the 35S::*GhSMT2–1*::NOS-T cassette; N, negative control, amplified from the genomic DNA of a non-transgenic cotton plant; W, blank control, amplified from a sample containing only water. **d** Relative expression level of the *GhSMT2–1* gene in the 10-DPA fibers and leaves of control and transgenic cotton plants. Total RNA was isolated from the 10-DPA fibers and leaves of control plants and transgenic lines. The first strand cDNA (from total RNA) was used as the template for RT-qPCR, using cotton *HISTONE3* for normalization of the data. OS-1 to OS-10 represents transgenic plant no. 1 to 10

In order to modify phytosterol composition, especially, the ratio of campesterol to sitosterol in cotton fiber cells, we plan to overexpress *GhSMT2–1* in cotton plant. A plant expression vector was constructed, in which the *GhSMT2–1* was under the control of CaMV 35S promoter (Fig. 1a). By genetic transformation on upland cotton, we obtained ten regenerated plants with kanamycin resistance. Since there is an expression cassette for *GUS* gene in T-DNA region, the kanamycin resistant plants were further confirmed by GUS staining (Fig. 1b). All plants were further characterized by amplifying the fragment of *GhSMT2–1* cDNA in transgenic cotton genome DNA (Fig. 1c). Finally, we detected the transcript abundance of *GhSMT2–1* in transgenic fiber cells by qRT-PCR. The result showed that the expression levels of *GhSMT2–1* increased compared to the control, indicating we obtained transgenic cotton plants overexpressing *GhSMT2–1* (Fig. 1d). According to the expression levels, two lines OS-6 and OS-9 were selected to further investigation.

### *GhSMT2–1* overexpression increased sitosterol and stigmasterol content and decreased campesterol content

**Fig. 2:**
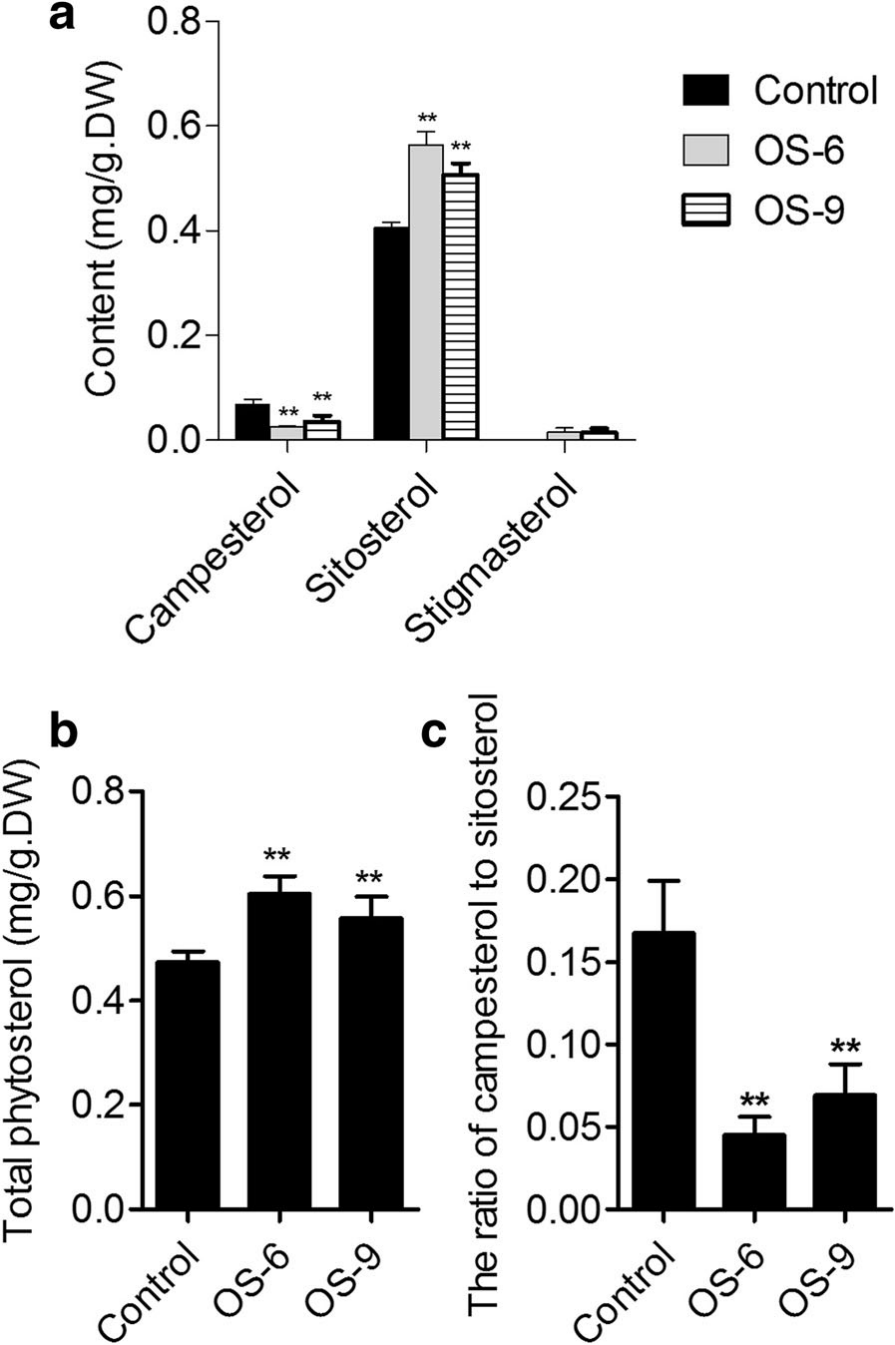
Change of phytosterols content and composition in transgenic 10-DPA fibers. 10-DPA cotton bolls were harvested and fibers derived from the ovule were rapidly dried at 70 °C. The dried material was ground to powder and quantified. 2.0 g 10-DPA fiber powder was used for phytosterol extraction. **a** Contents of campesterol, sitosterol, and stigmasterol in 10-DPA fibers from control, OS-6, and OS-9 plants. Error bars represent the SD for three independent experiments. **b** Total phytosterols in 10-DPA fibers from control and transgenic plants. **c** The ratio of campesterol to sitosterol in 10-DPA fibers from control, OS-6, and OS-9 plants. Asterisks indicate statistically significant differences between transgenic lines and the control, as determined by Student’s *t*-test (**, *P* < 0.01)

To characterize how to change phytosterol composition in transgenic cotton fibers, three phytosterols, sitosterol, stigmasterol, and campesterol were examined in 10-DPA fiber cells of OS-6, OS-9 lines, and control plants. In OS-6 fibers, the content of campesterol, sitosterol, and stigmasterol was 0.025 ± 0.002 mg/g. DW, 0.564 ± 0.025 mg/g. DW, and 0.015 ± 0.007 mg/g. DW, respectively. In OS-9 fibers, there are 0.035 ± 0.012 mg/g. DW campesterol, 0.507 ± 0.023 mg/g. DW sitosterol, and 0.015 ± 0.007 mg/g. DW stigmasterol. Compared to control fibers, campesterol was decreased by 62.5 and 48.4% in OS-6 and OS-9 fibers, respectively, while sitosterol was increased by 40.0 and 25.0% in OS-6 and OS-9 fibers, respectively. Furthermore, Stigmasterol was hardly detectable in 10-DPA fibers of the control plant, but a trace amount was detected in both transgenic plants (Fig. 2a). Concomitantly, the total content of phytosterols increased while the ratio of campesterol to sitosterol strikingly decreased in transgenic fiber cells when compared to the control fibers (Fig. 2b and c). These results indicated that overexpressing *GhSMT2–1* altered phytosterol content and composition, as well as the ratio of campesterol to sitosterol in transgenic fiber cells.

### *GhSMT2–1* overexpression shortened fiber length and thickened secondary cell wall

**Fig. 3:**
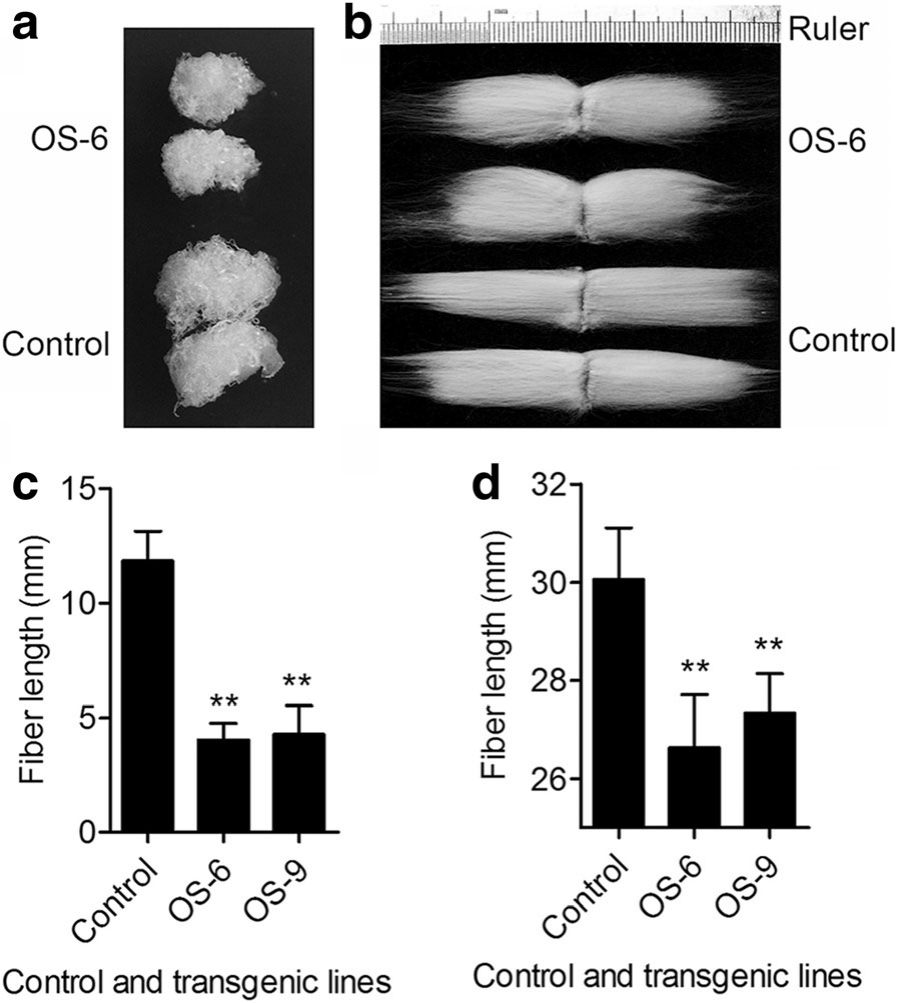
Fiber length in transgenic lines and the control. a and c is the photo and fiber length data from cotton ovule culture in vitro, respectively. The photo was taken before combing and showed the original shape of fiber (with ovule) after culture. b and d is the photo and fiber length data of mature fiber from plant, respectively. The photo was taken after combing. **a** The fibers (with ovules) of the control and transgenic lines after 10-day culture. **b** Mature and dried fibers (combed by hand before photographing) from the control plant and transgenic plant OS-6. **c** Fiber lengths of control and transgenic cotton lines after 10-day culture; error bars represent the SD for at least 15 seeds. **d** Lengths of mature fiber from control and transgenic lines. OS-6 and OS-9: transgenic lines 6 and 9 overexpressing *GhSMT2–1*. Error bars represent SD for at least 20 seeds. Statistically significant differences based on paired Student’s *t*-test at *P* < 0.01 are denoted by double asterisks

To understand the effects of phytosterol composition modification on fiber cell growth, we investigated fiber length and cell wall thickness when the fibers matured. The length of transgenic fibers was shorter than that of control fibers (Fig. 3b). The fiber length of OS-6 and OS-9 were 26.6 ± 1.1 mm and 27.3 ± 0.8 mm, respectively, while the control fiber was 30.2 ± 1.7 mm. The transgenic fiber was 11.9–9.6% less than that of the control (Fig. 3d). To further confirm the inhibitory effect on fiber elongation, the ovules from OS-6, OS-9, and control plants were cultured in vitro. The transgenic fiber growth was found to be inhibited when compared to control fibers (Fig. 3a). After 10-day culture, the lengths were 11.8 ± 1.3 mm, 4.1 ± 0.7 mm, and 4.3 ± 1.3 mm of control, OS-6, and OS-9 fibers, respectively. The OS-6 and OS-9 fiber lengths were 65.8 and 63.8% less than that of the control, respectively (Fig. 3c). These results indicated that the elongation of transgenic fiber cells was suppressed.

**Fig. 4:**
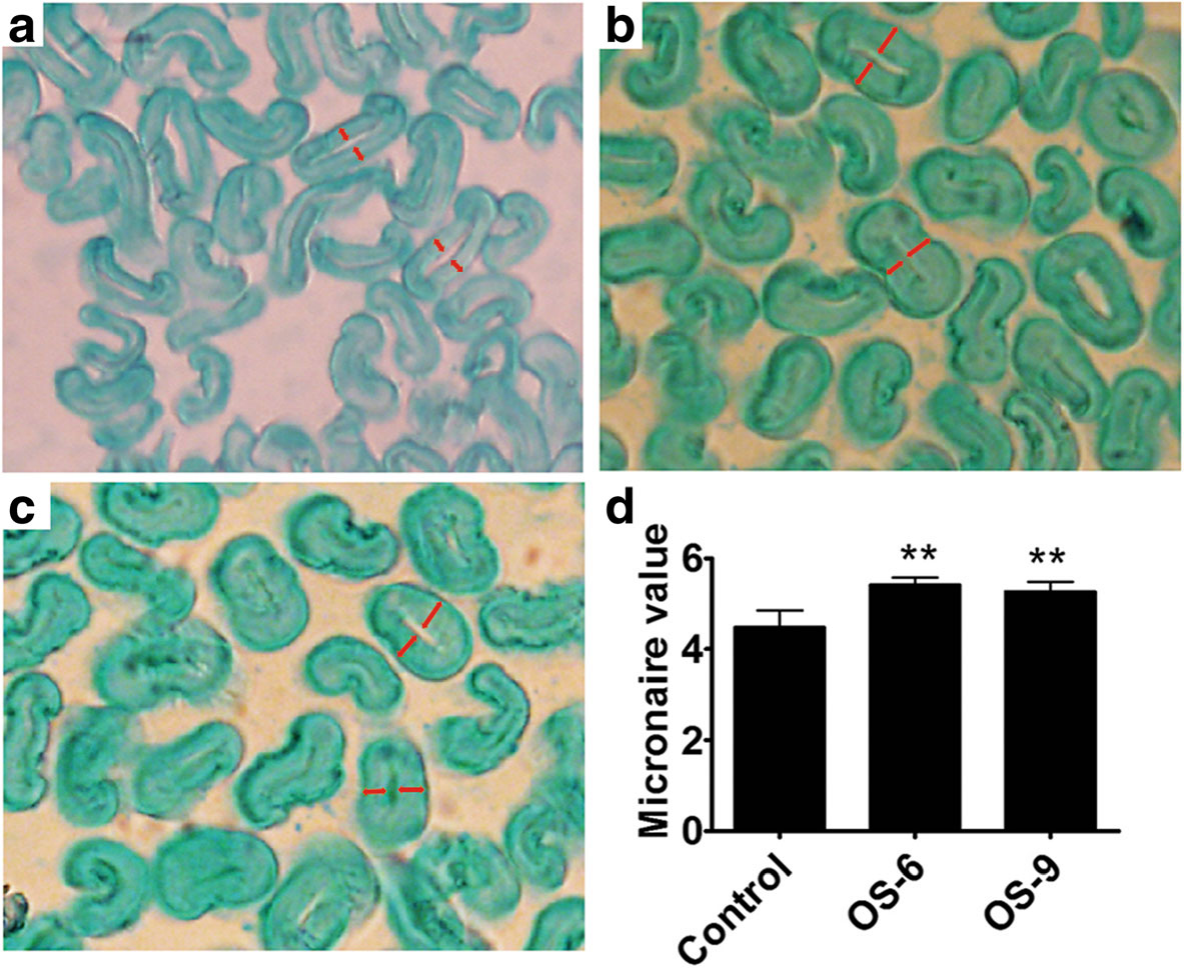
Micronaire values and secondary cell wall thickness of mature fiber. **a**-**c** Microscopic images of cross-sections of mature fibers of the control (**a**), transgenic OS-6 (**b**), and OS-9 (**c**). The thickness of secondary cell wall is indicated by a red demarcation. **d** Micronaire values of control and transgenic mature fibers. Error bars represent the SD of three biological replicates. Statistically significant differences based on paired Student’s *t*-test at *P* < 0.01 are denoted by double asterisks

We investigated the thickness of the secondary cell walls of mature fibers from transgenic plants and the control plants. The secondary cell walls of the OS-6 and OS-9 fibers were thicker than those of the control fiber (Fig. 4a–c). Micronaire value is a composite measure of fiber fineness. We further found that the micronaire values of OS-6 and OS-9 were significantly higher than that of the control (Fig. 4d). The results indicated that the thickness of fiber secondary cell wall in transgenic cotton plants increased.

Taken together, these results revealed that altering phytosterol composition influenced fiber elongation and secondary cell wall deposition.

### Exogenous application of sitosterol suppressed fiber elongation in vitro

**Fig. 5:**
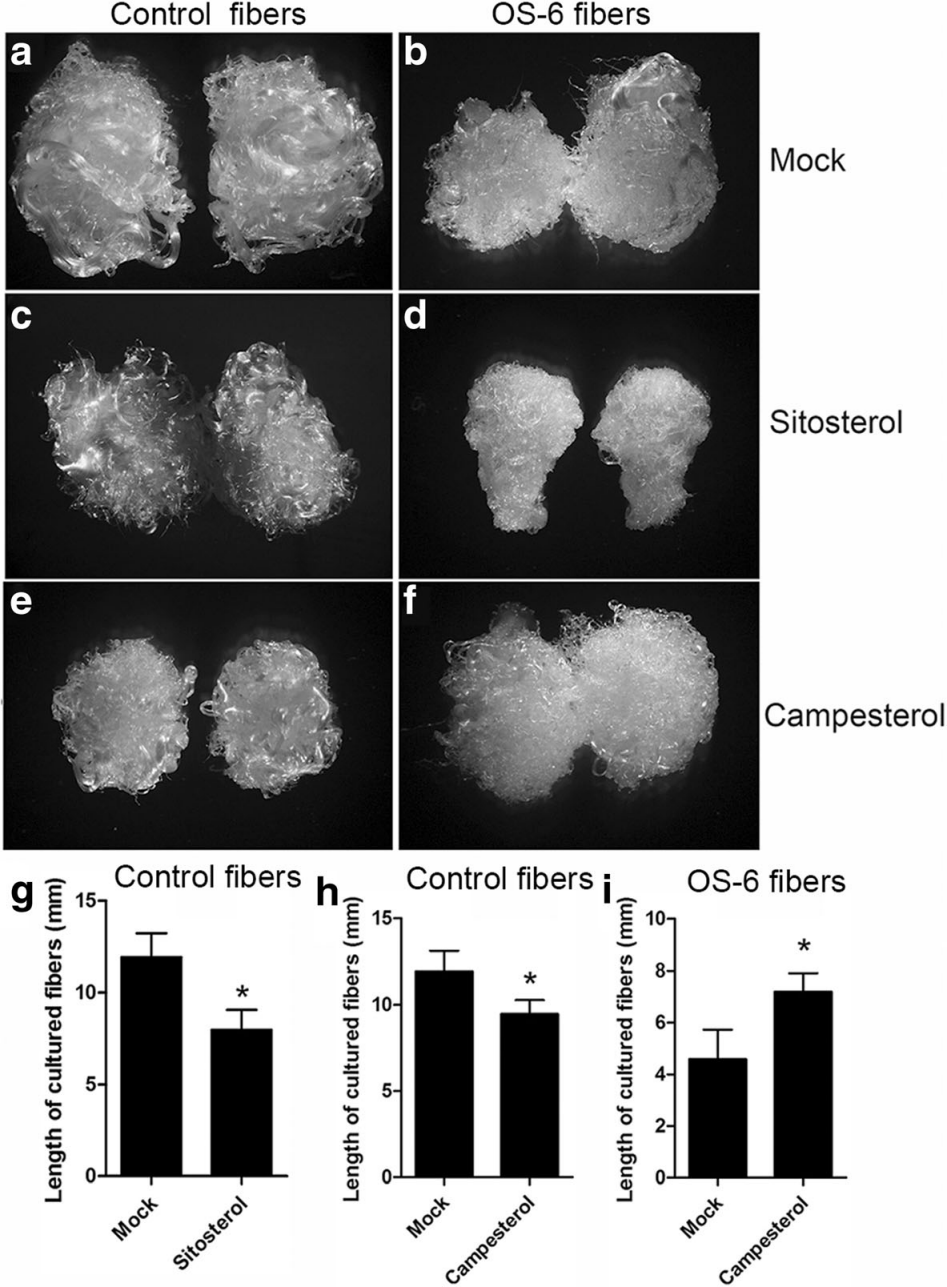
Effects of sitosterol or campesterol on fiber growth in ovule culture system in vitro. a and b Mocks for the ovules from control plants (**a**) and transgenic cotton lines (**b**). BT medium adjusted with the amount of ethanol equivalent to that used to dissolve sitosterol or campesterol was used as the mock. **c** and **d** Sitosterol treatment of fibers from control plants (**c**) and OS-6 line(**d**). **e** and **f** Campesterol treatment of fibers from control plants(**e**) and OS-6 line (**f**). All ovules derived from control and transgenic plants were cultured on liquid BT medium for 10 days. **g** Fiber length of mock and sitosterol-treated ovules of the control after 10-day culture in BT medium. **h** Fiber length of mock and campesterol-treated ovules of the control after 10-day culture in BT medium. **i** Fiber lengths of mock and campesterol-treated ovules of the OS-6 transgenic plant after 10-day culture in BT medium. Error bars represent SD for at least 10 seeds. Asterisks indicate statistically significant differences between the mock and treatment, as determined by Student’s *t*-test (*, *P* < 0.05)

Our results showed that sitosterol overproduction was in transgenic short fibers. To determine whether sitosterol alone had such an impact, exogenous sitosterol was applied for culture fibers. The fiber length of wild-type ovules cultured on medium containing 1.0 μM sitosterol was 8.0 ± 1.1 mm, which was much shorter than that of the mock (11.9 ± 1.3 mm) (Fig. 5a, c, and g). Meanwhile, exogenous application of sitosterol also conspicuously inhibited the growth of fibers from transgenic cotton plants (Fig. 5b and d). These results indicated that increased sitosterol suppressed fiber cell elongation.

### Exogenous application of campesterol suppressed fiber elongation on wild-type ovules

Campesterol content declined in transgenic fibers, which could possibly explain the fiber length decrease although the reduction amount is slight. To test this hypothesis, exogenous campesterol was applied for cultured fibers. The length of cultured fibers was 9.5 ± 0.8 mm when the medium contained 1.0 μM campesterol, which was 20.4% lower than the length of mock-treated fibers (Fig. 5a, e, and h). However, transgenic fibers were longer than mock fibers when campesterol was added (Fig. 5b, f, and i). The length of treated OS-6 fibers increased by 57.0% when compared to the mock (Fig. 5i). These results indicated that campesterol application, at a certain degree, suppressed fiber cell elongation in control, but rescued transgenic fiber elongation.

## Discussion

As a precursor of plant steroidal hormones (BRs) and a membrane component, phytosterol has a lot of functions in the growth and development of plant cell [20–26]. Cotton fiber is a highly elongated single cell, which is regarded as an ideal material for studying plant cell growth. Nevertheless, little is known what effect of phytosterols on fiber cell growth. In previous study, we have measured the content of three predominant phytosterols: sitosterol, campesterol, and stigmasterol in fiber cells at stages of elongation and secondary cell wall deposition. The phytosterol content and the ratio of campesterol to sitosterol were associated with fiber elongation and cell wall formation [4]. However, it is unclear what effect of content and/or composition change on fiber growth. To address this question, we firstly generated transgenic cotton plants in which the *GhSMT2–1* was overexpressed. The result was consistent with previous reports that total phytosterol content and sitosterol increased while campesterol and the ratio of campesterol to sitosterol decreased when compared to control [36, 37, 39, 40]. It suggested that GhSMT2–1 is a functional sterol C-24 methyltransferase 2, and overexpression using the CaMV 35S promoter in cotton results in transgenic fibers with altered phytosterol content and composition.

According to the high expression level of *GhSMT2–1* and the high content of sitosterol in fiber cells at elongation stage [4, 41], we expected that overexpressing *GhSMT2–1* would promote fiber cell elongation. However, the transgenic fiber cell reduced length either in plant or in vitro. High-sitosterol *Arabidopsis* plants have an overall reduced stature, indicating a defect in cell elongation [37]. In our current study, the elongation of high-sitosterol fiber cell also was suppressed, which resulted in short fiber in transgenic plants. Differently, the high-sitosterol fiber cell has thicker secondary cell wall than control. This phenotype was not reported in high-sitosterol *Arabidopsis* or tobacco plants [37, 39]. On the one hand, phytosterol may play a role in cellulose biosynthesis and cell wall formation [23, 42]. It was shown that sitosterol-beta-glucoside can serve as a molecular primer for cellulose synthesis in a reconstituted system [21]. The *cvp* mutants (that affect function of SMT2) display defects in vascular cell polarization and axialization in cotyledons [21, 35]. Furthermore, exogenous application of sitosterol up-regulated the expression of genes involved in cell wall development [43]. For example, *KOR*, encoding a membrane-bond endo-β-1, 4-glucanase, is critical for cellulose synthesis, cell wall assembly, cell expansion, and cytokinesis [21, 43–45]. Therefore, high-sitosterol might promote cell wall formation in fiber cells. On the other hand, there is partial overlap between fiber elongation and secondary cell wall deposition. Advanced secondary cell wall deposition must shorten the period of fiber elongation and accordingly prolong the period of cell wall deposition, resulting in shorter and thicker fibers. The fluctuation tendency of two major phytosterols at secondary cell wall deposition stage is sitosterol increase and campesterol decrease following fiber growth, which is similar with the pattern of two phytosterols in 10-DPA transgenic fibers, suggesting secondary cell wall might occur in 10-DPA fiber cell of transgenic plants.

Campesterol decreased in transgenic fibers. To examine whether campesterol defect resulted in short fiber, we conducted exogenous application of campesterol on transgenic and wild-type ovules in ovule culture system. Campesterol treatment rescued transgenic fiber elongation at a certain degree while suppressed wild-type fiber elongation, which is similar with the growth reduction in high-campesterol plants (SMT2:1 co-suppression) [37]. It suggested that campesterol application did not promote fiber elongation directly, only rescued the balance between sitosterol and campesterol in transgenic fiber cell. It is difficult to illuminate the role of campesterol or sitosterol independently since two molecules were linked tightly through SMT2 in plant. Schaeffer et al. proposed a role of SMT2 in balancing the ratio of campesterol to sitosterol to fit both growth requirements and membrane integrity [35, 37]. Our study, at a certain degree, confirmed this hypothesis. Additionally, specific phytosterol composition may be a signal involved in secondary cell wall formation.

Campesterol is a precursor of BR biosynthesis and one of the predominant phytosterols in plant. It is possible that altering phytosterols could affect BR biosynthesis. However, growing evidence indicated that sterols have regulatory roles in plants, independent of their contribution to BR biosynthesis. Carland et al. summarized the difference in role between phytosterol and BR. First, the phenotypes of sterol biosynthetic mutants are distinct from those in the downstream BR biosynthetic pathway. Second, transcriptional profiling experiments indicated that different sets of downstream genes are affected in sterol and BR pathway mutants. Third, sterol mutants influence membrane structure and traffic. Fourth, the sterol biosynthetic genes are expressed in regions of active cell division and expansion [36]. To elucidate the roles of phytosterol and BR in the growth of cotton fiber cells, we have measured their contents in fiber cell at various developmental stages. Results showed that the levels and dynamics of phyotsterol accumulation were distinct from BRs in fiber cell [4]. Campesterol was about 0.076, 0.028, and 0.010 mg/g. DW in 10-, 20-, and 30-DPA fiber cell, respectively [4] (Additional file 1: Table S1) while 6-Deoxotyphasterol, the most abundant BR in fiber was 2.0, 1.9, and 2.1 ng/g. FW in 10-, 20-, and 30-DPA fiber cell, respectively (Additional file 1: Table S1). These results indicated that campesterol concentration is far higher than BR concentration in fiber cell at same developmental stage, and campesterol decreased gradually while BR was almost stable following fiber cell growth. Therefore, we presumed that campesterol decrease at a certain degree did not directly result in BR decrease. The transgenic fiber length decrease, in present study, might not result from BR decrease. This idea also was supported by the report that campesterol increase did not elevate BR level in *cvp1* mutants [36]. Indeed, as a component of cell membrane, phytosterols play important roles in regulating the properties of cell membrane such as fluidity and permeability. Experiments with model membranes composed of phosphatidylcholine (PC) and soybean sterols have shown that all of the plant sterols can regulate membrane fluidity, but with different efficiencies [46]. Thus, the intracellular balance of 24-methyl- and 24-ethylsterols is important for plant membrane functioning, while the regulation of compositions and ratios of different types of sterols ensures the efficient implementation of membrane-associated processes [46]. BRI1, a BR receptor is a transmembrane protein. To further explore the relationship of phytosterol with BR, a future study might focus on the effect of phytosterol on BR signaling.

## Conclusion

The phytosterol content and composition of transgenic cotton fiber cells were modified by overexpressing *GhSMT2–1*, encoding a sterol C-24 methyltransferase. Accordingly, transgenic fiber length was shorter and secondary cell wall was thicker than that of control. Exogenous application of sitosterol or campesterol individually also suppressed fiber elongation. These results indicated that high-sitosterol and/or low ratio of campesterol to sitosterol suppressed fiber cell elongation and promoted secondary cell wall formation. Specific ratio might be a signal to trigger secondary cell wall deposition, and consequently, inhibited cell elongation.

## Methods

### Plant materials and growth conditions

The plant, used in this study was upland cotton (*Gossypium hirsutum* L.) cv. Jimian14 which was kindly provided by Hebei Agricultural University, China. Transgenic cotton plants were grown in the greenhouse under natural and additional artificial light (14 h light photoperiod at 150 μmol m^− 2^ s^− 1^) at 28–34 °C during the day and 24–27 °C at night.

### Vector construction and plant transformation

To construct a convenient plant expression vector, plasmid pBI121 was modified. The modified vector, named pBI121-GN, contained CaMV 35S promoter::*GUS*::nos and nos promoter::*NPTII*::nos fusion genes. To construct a sense *GhSMT2–1* cassette, the *GhSMT2–1* cDNA fragment was inserted into the pBI121-GN vector. The resultant vector, named pBI121-GN-OS, contained the sense *GhSMT2–1* sequence under the control of the CaMV 35S promoter (Fig. 1a). The pBI121-GN-OS plasmid was introduced into the *Agrobacterium* strain LBA4404. The transformation of upland cotton (*Gossypium hirsutum* L.) plants was performed using the pBI121-GN-OS vector according to a previously described method [7].

### Segregation analysis and homozygosity determination

Kanamycin-resistant seedlings were further confirmed by GUS staining. The segregation pattern of the selfed progeny (T1) and the homozygosity of T2 plants were also determined by GUS staining.

### Characterization of transgenic plants

Leaves from transgenic plants were examined for presence of the CaMV 35S promoter::GUS::nos gene using a histochemical assay for GUS activity. GUS-positive transgenic plants and wild-type control plants were grown in pots in a greenhouse under similar condition (28–35 °C by day and 20–25 °C by night with a 16/8 h light/dark cycle).

For molecular characterization of the transgenic lines, the genomic DNA isolated from transgenic and wild-type leaves was used as the template for PCR. Two primers were used: 35S-P1 (5′-TGGATCCGACCCTTCCTCTATATAAGG-3′), specifically recognizing the end of the CaMV 35S promoter, and GhSMT2-P2 (5′-CTGGAAGACAATGCAGCAAAT-3′), specifically recognizing the 3′-terminal of *GhSMT2–1* cDNA. The PCR thermocycling conditions were as follows: 94°C for 5 min; 30 cycles of 94°C for 30 s, 56°C for 30 s, and 72°C for 90 s; and 72°C for 10 min. Genomic DNA was isolated from young expanding leaves by plant genomic DNA traction kit (Aidlab Biotechnologies, China).

### RNA extraction and qRT-PCR

Total RNAs of 10-DPA fibers and leaves were isolated by the method described previously [47]. First-strand cDNA was synthesized from total RNA. Reverse transcription assays were carried out to synthesize the first strand of cDNA according to the instruction manual of the RT-PCR kit (TaKaRa, Dalian, China). In the real-time PCR system, the primer pair comprising P3 (5′-CACCATCAACGAGTACCAAGT-3′) and P4 (5′-CTGGAAGACAATGCAGCAAAT-3′) was used to detect *GhSMT2–1*. PCR was performed on a multicolor real-time PCR detection system (CFX96 Real-Time System; Bio-Rad, Hercules, CA, USA) with the following program: 94 °C for 2 min, followed by 40 cycles of 94 °C for 30 s, 56 °C for 30 s, and 72 °C for 1 min. Cotton *HISTONE3* (GenBank accession no. AF024716) was used to normalize data. The primers for *HISTONE3* PCR are HIS3-up (5′-GAAGCCTCATCGATACCGTC-3′) and HIS3-dn (5′-CTACCACTACCATCATGGC-3′).

### Extraction and dosage detection of Phytosterols

To extract the phytosterols of fiber cells, the method previously described by Deng et al. was used [4] . 10-DPA cotton bolls were harvested and fibers derived from the ovule were rapidly dried at 70 °C. The dried material was ground to powder. 2.0 g 10-DPA fiber powder was used for phytosterol extraction.

We performed GC-MS analysis using a gas chromatograph mass spectrometer (GCMS-QP2010S; Shimadzu, Kyoto, Japan) under the conditions previously described [4].

### In vitro ovule culture, Fiber length measurement, and Phytosterol treatment

In vitro cotton ovule culture and fiber length measurement were conducted as described previously [7]. Sitosterol or campesterol was dissolved in 100% ethanol to a final concentration of 1.0 μM in BT medium [48, 49]. BT medium adjusted with the amount of ethanol equivalent to that used to dissolve phytosterol was used as the mock.

### Analyses of Cell Wall thickness and Fiber Micronaire

We analyzed the cell wall thickness of mature fibers using the method previously described by Han et al. [50].

Fibers, after drying and ginning using a roller gin (SY-20, Jianghe Machinery Plant, Xinxiang, Henan, China), subjected to fiber micronaire value measurements at a HVI system (HFT 9000, Uster Technologies, Swiss) at the Center of Cotton Fiber Quality Inspection and Testing, Chinese Ministry of Agriculture (Anyyang, Henan, China).

## Additional file

Additional file 1: **Table S1**. The profiles of campesterol and three BRs in cotton fiber cell at various developmental stages. (DOCX 15 kb)

## Abbreviations

BRs: brassinosteroids
CaMV: Cauliflower mosaic virus
DPA: Day Post Anthesis
GhSMT2–1: a sterol C-24 Methyltransferase 2 of upland cotton (*Gossypium hirsutum* L.)
OS: transgenic cotton plants over-expressing *GhSMT2–1*
SMT2: Sterol C-24 Methyltransferase 2
