# Supplementary material for: Modification of phytosterol composition influences cotton fiber cell elongation and secondary cell wall deposition
Source: BMC Plant Biol. 2019 May 20;19:208. doi: 10.1186/s12870-019-1830-y (PMC6528235; doi:10.1186/s12870-019-1830-y)
Supplement: Supplementary file 1 — Table S1. The profiles of campesterol and three BRs in cotton fiber cell at various developmental stages. (DOCX 15 kb) [file 12870_2019_1830_MOESM1_ESM.docx]

Table S1. The profiles of campesterol and three BRs in cotton fiber cell at various developmental stages

| Developmental stage | 10 DPA | 20 DPA | 30 DPA |
| --- | --- | --- | --- |
| Campesterol (mg/g. DW) | 0.076 | 0.028 | 0.01 |
| 6-Deoxocathasterone (ng/g. FW) | 0.4 | 0.6 | 1.1 |
| 6-Deoxotyphasterol (ng/g. FW) | 2 | 1.9 | 2.1 |
| Typhasterol (ng/g. FW) | 0.3 | 0.1 | 0.1 |

*BRs were measured by Dr. Shozo Fujioka, RIKEN Advanced Science Institute, Japan)
